# Can High Throughput Phenotyping Help Food Security in the Mediterranean Area?

**DOI:** 10.3389/fpls.2019.00015

**Published:** 2019-01-25

**Authors:** Donatella Danzi, Nunzio Briglia, Angelo Petrozza, Stephan Summerer, Giovanni Povero, Alberto Stivaletta, Francesco Cellini, Domenico Pignone, Domenico De Paola, Michela Janni

**Affiliations:** ^1^Institute of Biosciences and Bioresources, National Research Council, Bari, Italy; ^2^Dipartimento delle Culture Europee e del Mediterraneo: Architettura, Ambiente, Patrimoni Culturali, Università degli Studi della Basilicata, Matera, Italy; ^3^ALSIA Centro Ricerche Metapontum Agrobios, Metaponto, Italy; ^4^Valagro SpA, Atessa, Italy; ^5^Institute for Veterinary and AgriFood Bioethics, Fiumicino, Italy; ^6^Institute of Materials for Electronics and Magnetism, National Research Council, Parma, Italy

**Keywords:** high throughput phenotyping, digital biovolume, water use efficiency, biostimulants, genetic resources, durum wheat, tomato

## Abstract

According to the IPCC 2014 report the Mediterranean region will be affected by strong climatic changes, both in terms of average temperature and of precipitations regime. This area hosts some half a billion people and the impact on food production will be severe. To implement a climate smart agriculture paradigm and a sustainable increase of agricultural productivity different approaches can be deployed. Agriculture alone consumes 70% of the entire water available on the planet, thus the observed reduction of useful rainfall and growing costs for irrigation water may severely constrain food security. In our work we focused on two typical Mediterranean crops: durum wheat, a rainfed crop, and tomato, an irrigated one. In wheat we explored the possibility of identifying genotypes resilient to water stress for future breeding aims, while in tomato we explored the possibility of using biostimulants to increase the plant capacity of using water. In order to achieve these targets, we used high throughput phenotyping (HTP). Two traits were considered: digital biovolume, a measure based on imaging techniques in the RGB domain, and Water Use Efficiency index as calculated semi-automatically on the basis of evaporation measurements resulting in a high throughput, non-destructive, non-invasive approach, as opposed to destructive and time consuming traditional methods. Our results clearly indicate that HTP is able to discriminate genotypes and biostimulant treatments that allow plants to use soil water more efficiently. In addition, these methods based on RGB quality images can easily be scaled to field phenotyping structure USVs or UAVs.

## Introduction

The agricultural sector is going to face enormous challenges in order to feed the 9.6 billion people that are going to inhabit the planet by 2050 (Elbehri, 2015). This goal has to be achieved in spite of limited availability of arable lands, of increasing need for irrigation water (agriculture consumes 70% of the world’s fresh water supply) and of the severe impact of climate change. It impacts on agriculture mostly through three drivers: water, heat and pests. Water plays an important role, due to its use for agriculture and also for most human activities. Drought affects a significant proportion of the global population, particularly those living in semi-arid and arid zones of the world, by increasing frequency, severity and duration of the adverse events (Steduto et al., 2012). The Mediterranean region has been indicated as one of the most prominent hotspots where the oncoming climate change will strike harder, with unpredictable impact on crop production in this area (Araus and Cairns, 2014; Reynolds et al., 2016). Recent years events in this region have demonstrated that drought stress (DS) severely impacts on wheat yield when occurring at grain filling stage (Berger et al., 2010) or during flowering and fruit enlargement in tomato (Jangid and Dwivedi, 2016). Periods of drought vary in timing and intensity and water is not used with equal efficiency during all crop stages (Reynolds et al., 2016). Drought hampers crop production and food security altering the photosynthetic efficiency, the nutrient uptake and the efficiency plants use water. Effects on plant growth under drought conditions are due to impaired enzyme activities, loss of turgor, and decrease in energy supply (Farooq et al., 2012). Climate change threats on wheat cultivation is even intensified by the great genetic uniformity of this crop in developed countries. In fact, wheat production for industrial food making generally relies on few cultivated varieties closely related to each other and genetically uniform (Lopes et al., 2015). These occurrences have increased the efforts of using in breeding schemes, the reservoir genetic diversity present in germplasm collection to identify traits able to mitigate the effects of climate change on crop production (Pignone and Hammer, 2013; Pignone et al., 2015). Recently climate smart agriculture (CSA) has been proposed as a further approach for developing actions needed to transform and reorient agricultural systems to effectively support development and ensure food security under climate change (du Jardin, 2015). Innovative agricultural practices are estimated to mitigate drought effects on crops and among them, a promising approach is the application of biostimulants at proper plant developmental stages (du Jardin, 2015); some experiences have in fact demonstrated the increase of plant tolerance to DS after biostimulant application (Petrozza et al., 2014; Rouphael et al., 2018) by improving leaf pigmentation, photosynthetic efficiency, leaf number and area, shoot and root biomass, as well as fruit number and/or mean weight, especially under adverse environmental conditions (Ertani et al., 2013, 2014; Petrozza et al., 2014; Colla and Rouphael, 2015; Lucini et al., 2015; Rouphael et al., 2018). In both approaches (plant breeding or new agricultural practices), intense experimental plant phenotyping is required to study and assess plant resilience to stresses.

While genomic tools are in place for major crop species giving a huge amount of data, the systematic quantification of phenotypic traits or components remains a big challenge (Chen et al., 2014). Thus, bridging the gap from genotype to the phenotype is one of the most important problems in modern plant science (Ubbens and Stavness, 2017). High-throughput phenotyping (HTP) has unlocked new perspectives for non-destructive phenotyping of large populations over time (Singh et al., 2016). It employs the acquisition of digital phenotypic traits by means of sensors, typically in the visible spectrum, as well in the near infrared, and in the induced fluorescence domain (Tardieu et al., 2017), to monitor the plants photosynthetic activity (Li et al., 2014; Fahlgren et al., 2015; Perez-Sanz et al., 2017), growth status (Pieruschka and Poorter, 2012; Petrozza et al., 2014) and the overall water content (Chen et al., 2014) as main components of plant response to limited water availability and heat stress (Comastri et al., 2018).

This paper aims at discussing two case studies, to demonstrate how high throughput phenotyping techniques may help promote the food security giving a special attention to the Mediterranean area by: (a) selecting new drought tolerant wheat genotypes from germplasm collections, and (b) increasing the understanding of the physiological mechanisms activated by the application of new biostimulant molecules able to improve water use in crops.

## Materials and Methods

### Plant Material, Growth Conditions, and Treatments

#### Wheat

A selection of durum wheat genotypes was grown in the Greenhouse at the Research Center Metapontum Agrobios (ALSIA). The germplasm panel comprised a set of 36 durum wheat genotypes, selected from a core set of 452, named SSD collection, produced by single seed descent from a worldwide durum wheat germplasm collection (Pignone et al., 2015). In recent years, the whole core set had been analyzed based on a set of morphometric parameters recorded in the RGB (Red, Green, Blue) domain and elaborated for morphological convolution. This analysis led to the identification of the 36 genotypes used in the present study that are highly representative of the variation of response to DS in the entire SSD collection. Three reference Italian varieties (Svevo, Saragolla, and Cappelli) were used as controls, considering their different response to drought (Table 1).

**Table 1:** SSD entry names and origins of the durum wheat genotypes used in the study.

SSD Entry	Origin
35	Algeria
44	Tunisia
64	Morocco
69	Morocco
92	USA- North Dakota
96	Azerbaijan
99	Ethiopia
109	Iraq
112	Iraq
116	Iraq
122	USA- North Dakota
135	Turkey
171	Peru
178	France
195	Saudi Arabia
231	Ethiopia
244	Ethiopia
253	Cyprus
269	Iran
278	Bulgaria
322	Turkey
325	Syria
335	Iraq
343	Iran
397	Crete
409	Greece
415	Crete
416	Greece
441	Crete
451	Iraq
459	USA
467	Greece
477	Italy
487	Greece
494	Greece
511	Lybia
Cappelli	Italy
Saragolla	Italy
Svevo	Italy

Seeds were germinated at room temperature for maximum of 4 days on moist filter paper in Petri dishes and then transplanted into polystyrene plateaus. The plateaus were then stored at 4°C for 2 weeks in order to synchronize the plantlets growth. Individual plants were then transferred into two liter pots filled with a 1:1(v/v) mixture of river sand and peat moss until a total weight of 1200 g. Then plants were grown in glasshouse under natural light conditions, and environmental conditions were monitored every 30 min using a datalogger (Watchdog Model 450, Spectrum Tecnologies, Inc.).

Three treated and three control replicates of each accession were randomly distributed in greenhouse to minimize through spatial distribution the possible establishment of microclimatic variation in the greenhouse. To identify each single pot a barcode was applied in proper position to allow automatic reading of the plant identifier.

**FIGURE 1:**
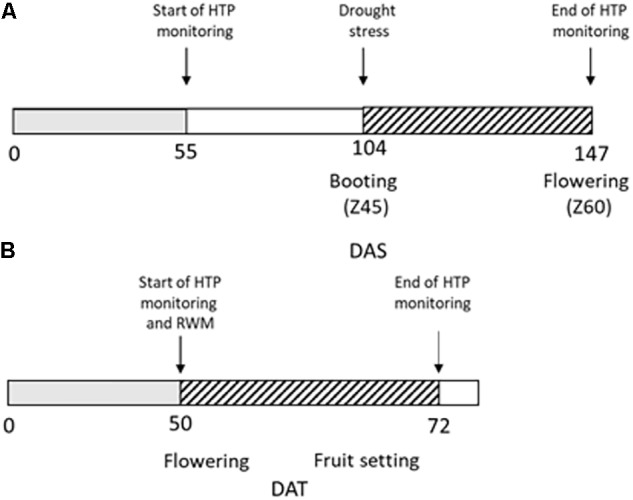
Experimental design of the high throughput phenotyping (HTP) experiment. **(A)** Durum wheat plants were regularly irrigated up to 104 DAS (days after sowing, gray box); drought stress (dashed box) was then imposed for 43 days from 104 to 147 DAS. RGB images for HTP were acquired from 55 DAS to 147 DAS; **(B)** Tomato plants were regularly irrigated up to 50 days from transplant (gray box) when reduced water management (RWM) was performed and biostimulants were applied three times in the 22 days indicated with the dashed box.

Wheat plants were manually kept fully irrigated up to the booting stage (Z45, 104 days after sowing, DAS), then DS was imposed for 43 days (up to 147 DAS) by maintaining the amounts of water in the soil around 50% field capacity (FC) through manual irrigation following pot weighting (Figure 1A).

#### Tomato

Thirty five tomato plants (*cv*. Ikram) were cultivated in 3 L pots filled with 1800 g of a substrate consisting of a 1:1(v/v) mixture of peat moss and river sand. The tomato plants were grown in glasshouse under natural light conditions. The environmental conditions were monitored every 30 min using a datalogger (Watchdog Model 450, Spectrum Tecnologies, Inc.).

At the moment of transplanting the plants were fertilized with 20 units of nitrogen, 40 units of phosphate (P_2_O_5_) and 20 units of potassium oxide (K_2_O) per pot.

Fifty days after transplanting, tomato plants were subjected to a reduced water management for 22 days (Figure 1B): the group of control plants UTC(70) was watered to restore 70% of the field capacity (FC), while another group of plants was kept at the same water regime and treated with six different natural biostimulant formulations (blindly identified as n. prototypes: 2148, 2197, 2219, 2220, 2221, and 2390). Biostimulant treatments were supplied in three applications by drenching, at the rate of 20 L/ha. Such biostimulants were particularly conceived (Valagro SpA) to improve water use efficiency (WUE) in different crops, including tomato. Their composition is proprietary, and the different formulation codes were provided by Valagro SpA for testing.

As control, 10 pots filled with the same soil mixture but without plants were randomly loaded on the conveyor belt LemnaTec system in order to estimate the daily water evaporation from bare soil.

### Phenotyping Analysis

Images in the RGB domain (white light) for HTP were captured every other day according to Petrozza et al. (2014), by using a Scanalyzer 3D system (LemnaTec GmbH, Aachen, Germany) held in the Phen-Italy platform located in ALSIA Metapontum Agrobios (Metaponto).

Several traits can be recorded by using the RGB module of the 3D Scanalyzer; each plant is imaged sequentially employing different wavelengths in the visible and non-visible spectrum even if for this investigation only the visible wavelength have been considered.

The imaging involving three mutually orthogonal vantage points was used to evaluate morphometric parameters of the plant, such as height, width, or biomass (Petrozza et al., 2014).

The digital biovolume (DB), was calculated from the three orthogonal images of the same plant according to the formula

(1) $$\sum \mathrm{pixelsideview}0{}^{\circ}+\sum \mathrm{pixelsideview}90{}^{\circ}+\log\left(\sum \frac{\mathrm{pixeltopview}}{3}\right)$$

(Eberius and Lima-Guerra, 2009) and is assumed to be proportional to the aerial mass of the plant (Poiré et al., 2014). The Digital Biovolume Ratio was calculated as (biovolume of treated plants/biovolume of control plants). Wheat plants were monitored applying image acquisition at 2-day intervals from 55 DAS up to 147 DAS for a total of 92 days, while tomato plants were monitor applying the same interval for a total of 22 days.

### Water Use Efficiency Estimation

Water use efficiency was determined for tomato plants and for all wheat genotypes using the following formula:

(2) $$\text{WUE}=\frac{{\text{DB}}_{{\text{t}}_{\text{n}}}-{\text{DB}}_{{\text{t}}_{0}}}{\sum_{{\text{t}}_{0}}^{{\text{t}}_{\text{n}}}\left(\text{Tr}\right)}$$

where DB_t_n__ is the DB at a specific time point, DB_t_0__ is the DB at 0 DAT for both experiments and the denominator corresponds to the sum of the quantity of water consumed (i.e., transpiration) during the corresponding growth period (Richards, 1991).

The volume of water evapotranspirated by individual plants was calculated as the difference between the weight measurement at field capacity and the current weight.

The WUE ratio index was calculated dividing the WUE of drought stressed plants by WUE of control plants for each genotype throughout the experiment. On the basis of this index, a heatmap chart was generated including each genotype using the R package ggplot2 (Wickham, 2009).

The green color indicates a higher WUE ratio and refers to values ranging between 1.32 and 1, red color indicates a lower WUE ratio including values raging between 1 and 0.52.

### Statistical Analysis

Descriptive statistical parameters for DB and WUE were calculated on wheat phenotyping data, ANOVA analysis was performed with SigmaPlot ver.13 (Systat Software Inc.). The *F* test statistic, calculated as the ratio between estimated population variance between groups and estimated population variance within groups, was determined. To demonstrate the correlation between DB and fresh weight biomass harvested a Linear Correlation Analysis was performed (SigmaPlot ver.13, Systat Software Inc.). Phenotyping data from the tomato trial were analyzed using one-way analysis of variance (ANOVA), and the means were compared using the Duncan’s New Multiple Range Test (MRT; *p* < 0.05) employing the R package agricolae (Mendiburu, 2016).

### Data Availability

Phenotyping raw data and images are available upon request to the authors. Tables containing data used for the analyses but non-included in the text, are reported in the Supplementary Material.

## Results

### RGB High Throughput Phenotyping Image Analyses to Monitor Plant Stress Response

The RGB imaging index DB was used to monitor the phenotypic response to DS in wheat and to assess the benefits brought by the application of biostimulants in tomato plants under reduced water conditions. The effectiveness of this digital parameter as indicator of the plant biomass variation during plant growth has been previously described (Poiré et al., 2014).

Wheat and tomato plants were monitored over a period of 13 and 3 weeks, respectively, through a non-invasive, non-destructive, high throughput phenotyping platform. The activity regarded plants used as controls and others grown under drought/water limiting stress conditions.

#### Assessing Durum Wheat Landraces Response to Drought

The RGB index was chosen to monitor and evaluate the differences in growth limitation following DS in the set of durum wheat landraces was DB. No significant difference in the DB was observed between treated and untreated plants in the period from 55 to 104 DAS, corresponding to the well-watered cultivation period; in this same period differences in DB could only be observed among the analyzed genotypes as expected.

**FIGURE 2:**
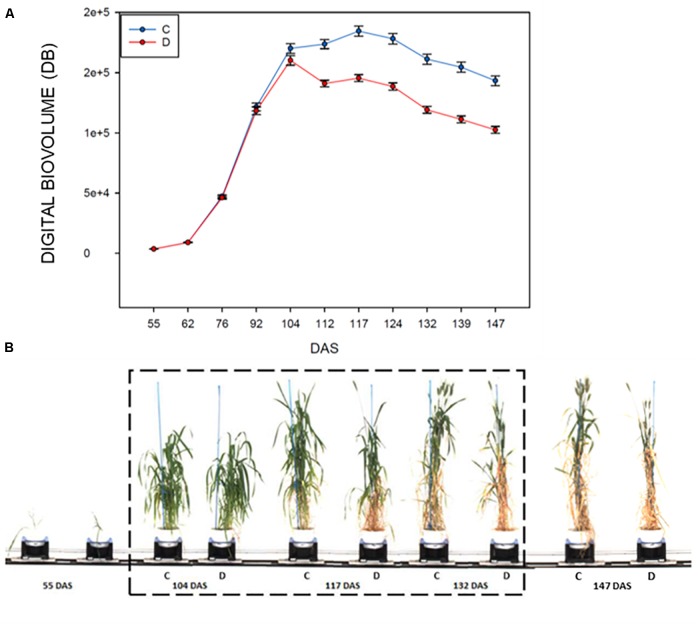
RGB HTP analyses for control and drought-stressed plants in the period 55–147 DAS, measured using a Lemnatec Scanalyzer 3D. **(A)** Mean value of the Digital biovolume (DB): C, control plant; D, drought stressed plants. **(B)** Example of the acquired RGB Images of a representative durum wheat genotype, showing the effects of drought stress on plant growth. The drought stress interval is indicated with dashed box.

The evaluation of the DB in control and drought stressed plants, showed that this index is significantly affected in plants subjected to drought starting at 8 days after the imposition of the stress (104–112 DAS, *F*178.181; *P* < 0.001; Figure 2A and Table 2), then it remained quite steady for 12 days, to drop significantly in the last 12 days of the withholding of water (124–147 DAS) reaching the minimum value recorded. The highest difference observed between the treatments was at 139 DAS (*F*351.417; *P* < 0.001, Figures 2A,B and Table 2).

**Table 2:** Digital biovolume average values of all the control and drought stressed SSD plants analyzed by HTP and results of the ANOVA test.

DAS	Digital biovolume		Treatment	Genotype	G × T
	C plants^∗^	DS plants^∗∗^			
55	3545.83	3594.66	*F* = 1.319; *p* = 0.253	*F* = 7.403; *p* < 0.001	*F* = 0.889; *p* = 0.655
62	9009.33	8876.71	*F* = 0.0472; *p* = 0.828	*F* = 14.366; *p* < 0.001	*F* = 1.023; *p* = 0.445
76	47345.44	46197.58	*F* = 1.935; *p* = 0.166	*F* = 18.241; *p* < 0.001	*F* = 1.296; *p* = 0.140
92	121543.46	118389.87	*F* = 3.708; *p* = 0.056	*F* = 20.059; *p* < 0.001	*F* = 1.915; *p* = 0.004
104	171139.38	160179.77	*F* = 10.299; *p* = 0.002	*F* = 18.862; *p* < 0.001	*F* = 0.824; *p* = 0.751
112	173693.21	140974.00	*F* = 178.181; *p* < 0.001	*F* = 17.754; *p* < 0.001	*F* = 1.333; *p* = 0.117
117	184458.11	145476.21	*F* = 242.848; *p* < 0.001	*F* = 21.295; *p* < 0.001	*F* = 1.675; *p* = 0.016
124	178176.04	138575.22	*F* = 235.265; *p* < 0.001	*F* = 22.406; *p* < 0.001	*F* = 2.071; *p* = 0.001
132	161236.76	119183.28	*F* = 301.286; *p* < 0.001	*F* = 24.497; *p* < 0.001	*F* = 2.736; *p* < 0.001
139	154549.81	111244.18	*F* = 351.417; *p* < 0.001	*F* = 26.295; *p* < 0.001	*F* = 2.645; *p* < 0.001
147	143304.76	102564.49	*F* = 303.089; *p* < 0.001	*F* = 22.379; *p* < 0.001	*F* = 2.369; *p* < 0.001

*F statistic represents the ratio between estimated population variance between groups and estimated population variance within groups. ^∗^C plants: control plants; ^∗∗^DS plants Drought Stressed plants.*

Generally, all the analyzed genotypes were affected by DS as variation in the DB. Nevertheless, the differences between the control and stressed lots of the same genotype changed significantly in the period of stress administration (104–147 DAS). Some genotypes were more affected by water limitation, while others appeared to be way more tolerant to the stress. In particular genotypes 44, 195, 269, 322, 409, 416, and 447 showed a smaller reduction in the DB during the entire stress period of the experiment (Table 3). Similar behavior was observed for the variety Svevo, generally considered a standard for tolerance to DS under Mediterranean agro-climatic condition, and the variety Cappelli, identified as source of drought resistance traits (Aprile et al., 2013).

**Table 3:** Digital Biovolume Ratio (DBR) calculated for all SSD genotypes tested and control varieties for the entire experiment length.

SSD	DAS										
	55	62	72	92	104	112	117	124	132	139	147
35	1.23	1.12	0.85	1.04	1.12	0.78	0.74	0.78	0.75	0.73	0.69
44	1.03	0.93	0.93	1.31	0.84	0.80	0.84	0.82	0.84	0.84	0.81
64	1.06	0.97	0.95	0.98	0.81	0.90	0.83	0.90	0.83	0.72	0.67
69	0.97	1.10	1.15	1.09	1.07	0.88	0.82	0.81	0.76	0.77	0.67
92	1.08	0.93	1.14	0.93	0.83	0.74	0.73	0.74	0.76	0.79	0.72
96	0.86	0.98	1.00	0.79	1.12	0.73	0.72	0.81	0.76	0.73	0.69
99	1.11	1.03	0.95	1.15	1.10	0.96	0.70	0.69	0.61	0.52	0.52
109	1.03	1.08	1.24	1.16	0.96	0.86	0.87	0.82	0.79	0.73	0.74
112	1.16	0.99	0.99	1.07	0.86	0.73	0.68	0.68	0.64	0.63	0.66
116	0.92	1.01	0.97	0.98	0.86	0.87	0.77	0.77	0.78	0.83	0.80
122	1.04	0.85	0.81	0.90	0.79	0.80	0.76	0.71	0.78	0.75	0.68
135	0.94	0.85	0.79	0.70	0.79	0.66	0.67	0.63	0.58	0.55	0.53
171	0.93	0.95	0.87	0.93	0.89	0.69	0.71	0.71	0.67	0.76	0.75
178	1.03	0.76	0.83	0.78	0.92	0.77	0.76	0.74	0.68	0.58	0.56
195	1.13	1.19	1.08	1.43	0.96	0.99	0.94	0.94	0.95	0.89	0.93
231	0.97	0.92	0.89	0.97	0.91	0.76	0.82	0.79	0.78	0.68	0.80
244	1.04	0.97	0.81	0.96	0.80	0.80	0.73	0.78	0.70	0.70	0.72
253	0.92	0.90	0.96	0.97	0.92	0.76	0.79	0.74	0.71	0.68	0.62
269	0.98	0.84	0.78	0.86	0.96	0.89	0.88	0.87	0.85	0.88	0.89
278	1.24	1.19	1.34	1.06	1.13	0.79	0.74	0.67	0.59	0.62	0.64
322	1.48	1.17	1.04	1.00	0.94	0.89	0.88	0.87	0.89	0.79	0.83
325	1.07	1.02	0.96	0.95	0.99	0.80	0.78	0.72	0.65	0.63	0.62
335	1.09	1.12	0.97	1.10	0.88	0.79	0.82	0.83	0.72	0.67	0.69
343	0.89	0.93	0.91	0.75	0.85	0.83	0.89	0.98	0.90	0.80	0.94
397	1.31	1.09	1.08	0.86	1.00	0.81	0.82	0.84	0.75	0.68	0.69
409	1.01	1.05	1.01	1.04	0.90	0.97	0.95	0.91	0.94	0.96	0.97
415	0.92	0.97	1.03	0.98	0.99	0.73	0.69	0.73	0.73	0.78	0.71
416	1.17	1.17	1.07	0.96	1.02	0.82	0.88	0.86	0.90	0.88	0.89
441	1.41	1.30	1.23	0.93	0.97	0.89	0.90	0.91	0.73	0.72	0.69
451	1.05	1.04	1.19	1.11	0.96	0.81	0.75	0.74	0.73	0.79	0.79
459	0.98	0.84	0.92	0.83	0.88	0.78	0.80	0.77	0.70	0.60	0.56
467	1.11	0.87	0.99	1.04	1.03	0.86	0.85	0.73	0.76	0.78	0.79
477	1.32	1.09	1.08	1.08	1.03	0.83	0.87	0.86	0.89	0.88	0.86
487	0.86	1.01	1.01	0.97	0.94	0.79	0.79	0.81	0.76	0.74	0.76
494	1.10	1.00	1.07	0.96	0.98	0.85	0.84	0.83	0.81	0.81	0.76
511	0.99	0.79	0.96	0.77	1.01	0.86	0.76	0.75	0.70	0.62	0.64
Cappelli	1.00	1.08	1.02	0.93	0.99	0.99	0.99	1.00	0.98	0.95	0.97
Saragolla	0.91	1.09	0.98	1.08	0.97	0.77	0.69	0.64	0.63	0.61	0.60
Svevo	0.89	0.89	0.88	0.69	0.93	0.93	1.03	1.01	0.97	0.90	0.88

*Digital biovolume ratio is calculated as: biovolume of drought stresses plants/biovolume control plants.*

#### Exploitation of Genetic Resources to Increase Water Use Efficiency

The WUE trend was studied in order to rank the genotypes according to their WUE, and to correlate this index with DS resilience. WUE was calculated as the above-ground biomass produced per mass of water consumed, including evaporation and transpiration (Richards, 1991) in the set of plants under measurement.

**FIGURE 3:**
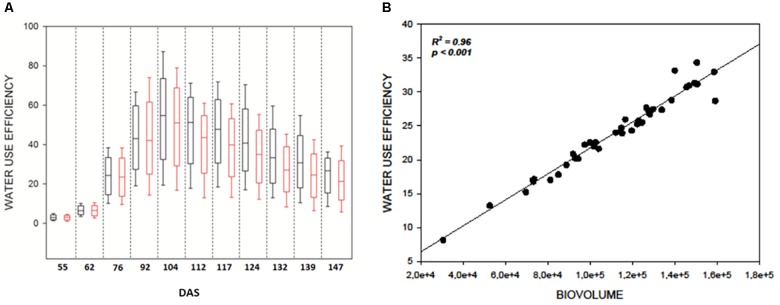
**(A)** Boxplots showing the distribution of WUE values during the experiment. Black boxes are control plants, red boxes the water stressed lot. Drought stress was imposed at 104 DAS; **(B)** Correlation plot between WUE index and digital biovolume acquired by Scanalyzer at 139 DAS.

As expected WUE was strongly affected by DS in all genotypes (Figure 3A) and significantly decreased immediately after the beginning of the stress at 104 DAS (*F*121.141; *p* < 0.001) (Figure 3A). It reached a minimum at 139 DAS (*F*231.910; *p* < 0.001) and remained constant till the end of the measurements (Supplementary Table 1). This result lead to hypothesize a direct correlation between the reduction of WUE in the plants and their reduction in biovolume as a consequence of the reduction of evapotranspiration in the stressed samples. The strong correlation (*R*^2^ = 0.96, *P* ≤ 0.001) observed between DB and WUE observed at 139 DAS (Figure 3B) supports this hypothesis (Figure 3B) and proves the efficacy of the DB as strong indicator of the plant health status.

**FIGURE 4:**
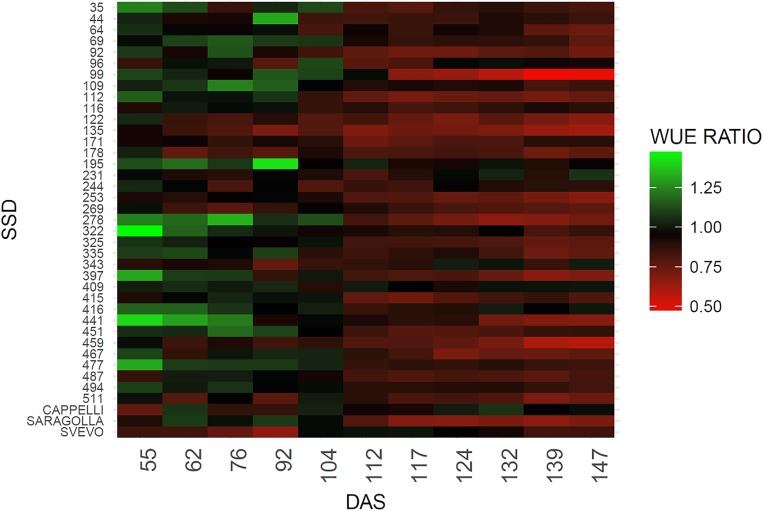
Heatmap of WUE ratio for the analyzed genotypes. The green color indicates a higher WUE ratio, red color a lower WUE ratio. WUE ratio is calculated as WUE of drought stresses plants/WUE control plants.

The WUE indicator also provides a measure of the different abilities to recover among different genotypes. To this purpose the WUE ratio (WUE drought/WUE controls) was estimated (Pandey, 2017) (Figure 4). As expected, a difference in the WUE ratio was observed among the genotypes due to the genetic variability posed in the collection. An overall reduction in the WUE was observed, however, SSD lines 69, 109, 195, 231, 244, 322, 343, 409, and 416 maintain similar values of WUE under stress condition for the entire set of measurements (Figure 4). The reliability of the measure is also supported by the observation that similar behavior is shown by the variety Cappelli reported to be drought resistant (Aprile et al., 2013). In contrast, SSD lines 99, 135, 253, 278, 397 and the Italian variety Saragolla showed a marked decrease in the WUE ratios immediately after the stress imposition (112 DAS), which remains of same entity for the entire experiment (Figure 4 and Supplementary Table 2). A high variability between SSDs was expected due to the high genetic diversity present in the material, however a certain range of variation in the WUE is also described by the heatmap before the stress imposition; this probably occurred for an operational bias in the manual watering procedures, thus confirming the high sensitivity of this method in fine recording small variation of WUE during the plant growth.

#### Impact of the Application of Natural Biostimulants on Digital Biovolume and Water Use Efficiency in Tomato Plants

**FIGURE 5:**
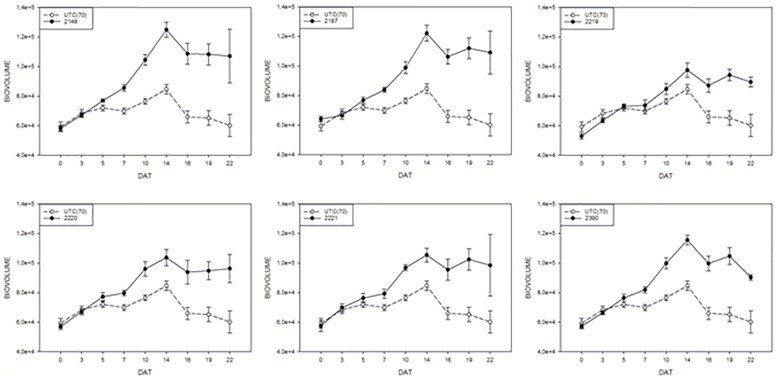
Digital biovolume (DB) measurements on tomato plants exposed to different biostimulant prototypes: UTC(70) refers to the Untreated Control thesis where plants were irrigated with 70% FC, indicated with a dashed line. Tomato plants under reduced water condition (70% FC) and treated with the biostimulant prototypes are indicated with the solid line.

In order to monitor the effect of biostimulants on tomato plants grown under limiting water availability, the variation of the digital biomass based on RGB imaging was used. The differences in DB value between the UTC(70) and biostimulants-treated plants were observed. Interestingly, all prototype formulations exerted a consistent increase in DB in comparison with UTC(70). In particular, prototypes 2148, 2197, and 2390 were the most effective (Figure 5); the observed increase in DB was consistent and significant already from 7 Days After Treatment (DAT, Figure 5) and reached a peak at 14 DAT, when the maximum difference between treated and untreated plants was recorded. The observed increase in DB values in the biostimulant-treated plants indicates an overall benefit of such formulations on plant growth and development, in particular under limited water management (Figure 5).

**FIGURE 6:**
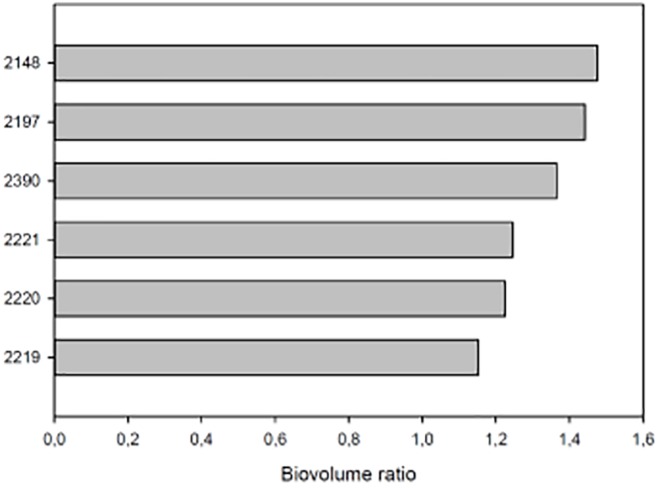
DBR at 14 DAT of tomato plants exposed to different water limiting conditions. The UTC(70) plants were used as reference. On the *Y* axis each bar is identified by the number of the prototype formulation used to treat tomato plants. On the *X* axis the biovolume ratio. All plants were growth at 70% of the field capacity (FC) and treated with the relative prototype as indicated in Section “Materials and Methods.”

A closer look at the 14 DAT time-point, when the highest difference in DB was observed between biostimulants-treated and UTC(70), allowed a ranking of biostimulant prototypes efficacy. To better express biostimulant performance, the digital biovolume ratio (DBR) was used. The ranking showed (Duncan’s MRT; *p* < 0.05) that prototype 2148 formulation was the most effective in increasing DBR (Figure 6).

**FIGURE 7:**
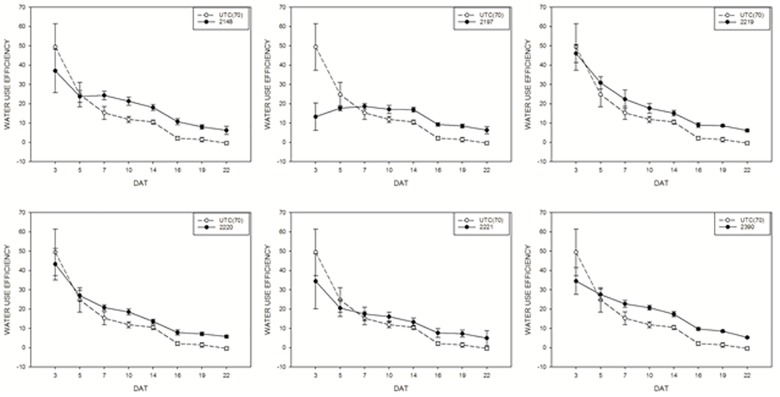
Water use efficiency mean values of tomato plants exposed to different watering conditions: UTC(70) refers to the Untreated Control thesis where plants were irrigated with 70% FC, indicated with a dashed line. Tomato plants under reduced water condition (70% FC) and treated with the biostimulant prototypes are indicated with the solid line.

Water use efficiency of tomato plants treated with biostimulants compared to UTC(70) control plants was also evaluated. Considering this parameter, an overall positive effect of the application of biostimulant prototypes was observed for all prototypes (Figure 7), in line with the observations on DBR. Prototype 2148 (Talete^®^), which was the most effective in increasing DB, was also able to increase consistently the WUE of plants under limiting water conditions, thus confirming this prototype as the best candidate for further commercial development.

### Is the Imaging Based Digital Biovolume Efficient in Determining Plant Growth and Biomass?

**FIGURE 8:**
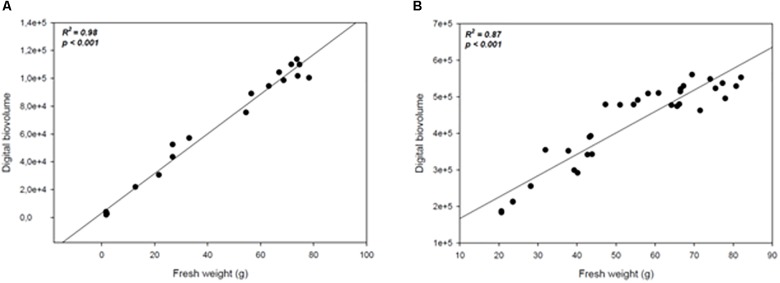
Scatter plots of the automated (digital biovolume) and manual (fresh weight) measurements in Wheat **(A)** and Tomato **(B)**. The scatter plots and linear regressions displayed in **(A,B)** indicate a strong correlation between the estimated and the manually measured parameters in wheat and tomato plants, with correlation coefficients of 0.98 and 0.87 respectively to the plants’ fresh weight.

In order to assess whether the image acquisition system provided a reliable representation of plants growth under drought conditions or upon treatment with biostimulants under reduced water availability, a measurement of plant biomass using traditional destructive methods was performed for both crops: at 147 DAS for wheat, and every 4 days throughout the experiment for tomato. The manually measured fresh weight was compared with the automated DB value previously acquired at the same time point. A high positive correlation (*R^2^* = 0.98; *P* < 0.0001; *R^2^* = 0.87; *P* < 0.0001) was recorded between the automated DB and the manual fresh weight in both wheat and tomato plants (Figures 8A,B). Our results are in agreement with previous reports (Poiré et al., 2014) thus validating the DB as an effective plant biomass predictive tool. It could be used in germplasm selection aimed at pre-breeding and breeding programs or in evaluating the effect of agricultural practices on plant growth.

Taken all together these results support the efficacy of the DB as a strong phenomic indicator of the overall health status of the plant following limiting growth conditions in both crops. The characteristic of being non-destructive, scalable and applicable to many crops plants enrich its applicability for both basic and applied research.

## Discussion

Recently the concept of Climate-smart agriculture (CSA) was proposed to increase the sustainability of agricultural systems, by reorienting the agricultural development to face the treats of climate change (Lipper et al., 2014). CSA aims at effectively supporting development and ensuring food security, particularly in those areas where climate change strikes, by achieving three main objectives: increasing sustainable agricultural productivity; adapting and building resilience to climate change; and adopting techniques that reduce greenhouse gas emissions (Food and Agriculture Organization [FAO], 2017). Most of the impacts of climate change on agricultural systems are expected to result from changes in the water cycle impacting on both rainfed and irrigated crops as a consequence of: increased evapotranspiration, changes in the amount and regime of rainfall, and variations in water availability from both surface and ground sources. Our paper focused on Mediterranean agriculture and two key crops of this region characterized by different water management: durum wheat, a rainfed crop that represents the principal food grain of the Mediterranean, and tomato, an irrigated crop being a key element of the Mediterranean diet and a cash crop of the area.

Two different climate smart strategies for the two crops have been identified: digging into the vast genetic pool of durum wheat in order to identify resilience traits for the next generation of varieties, and identifying agricultural practices aimed at improving the utilization of irrigation water in tomato.

To tackle these strategies, we have explored the potential of high throughput phenotyping technologies. Until recent, phenotyping has been hampered by the huge amount of human work needed to collect enough data. The recent introduction of high throughput phenotyping facilities has brought to researchers, breeders, and agriculturalist a bunch of new techniques to collect a paramount mass of data in a continuous, non-invasive, non-destructive way. The aim of this paper was to verify the possibility of utilizing a high throughput phenotyping infrastructure, located in Metaponto (Matera, Italy), to contribute to maintain food security notwithstanding the effects of climate change in the Mediterranean area.

We have used high resolution RGB images, taken in three projections by the platform, and elaborated through proper algorithms, in order to evaluate two parameters considered related to resilience to DS: DB and WUE. Both these parameters have been based on data recorded in a non-destructive manner, which allows to examine one and the same plant over the time.

The information on plants phenotype collected by RGB cameras provide a huge amount of high quality data, thanks to the high resolution of these images. The use of an RGB high quality camera has some advantages over multi- or hyper- spectral sensors. In fact, if on the one side vegetation indices formulated using RGB images have a more limited spectral range and color resolution (only broad bands within the visible region) with respect to the equivalent indices formulated by spectral sensors, on the other side RGB images have generally a much higher spatial resolution than spectral imagers. In this kind of analyses, high spectral resolution can be worthwhile substituted by high spatial resolution with equal or even better results (Araus and Kefauver, 2018).

In the present study we have exploit a digital biomass index as predictive of physical measurement of the plant biomass. The latter method, the traditional one, is based on weighing the cut plant and recording its fresh weight. This is a destructive method. High Throughput Phenotyping, on the contrary, is based on non-destructive methods since they allow the analysis of a single and the same plant over time, thus reducing the possible bias introduced by small individual variation. Moreover, this timescale phenotyping approach can even be used to unveil genetic traits that could not be otherwise analyzed using traditional methods (Busemeyer et al., 2013).

Water use efficiency based on an automatable process expressing the amount of water used by the plant from that evaporated from the soil was also evaluated. The effective measure of WUE is based on isotopic discrimination of Carbon (Seibt et al., 2008) which is a complicated and time consuming process. In this paper we use a digital extrapolated WUE with the idea that this is a parameter able to provide indication on drought resilience. In the Mediterranean region rainfed agriculture is facing unpredictable seasonal rainfall. Under these conditions the ability to use at best the soil moisture is a crucial component of drought resistance (Blum, 2005).

Further statistical analyses, such as regression analysis, support the efficacy of high throughput phenotyping in monitoring plants WUE, and highlight how DB can be used as an index to perform quality testing on the efficacy of biostimulant formulations in improving plant performance under reduced irrigation regimes. The latter observation is in line with the view of the European Biostimulant Industry Council (EBIC, 2018), that promotes the use of plant biostimulants to foster plant growth and development throughout the crop life cycle in a number of demonstrated ways, including the improvements of WUE (EBIC, 2018).

The development of effective HTP platforms, especially open field HTP facilities, is a bottleneck for future breeding progress (Araus and Cairns, 2014). The test of analytical approaches under controlled conditions is essential to devise and implement technologies to be transferred to the field.

So far, HTP platforms employ a variety of imaging methodologies to collect data for quantitative studies of complex traits related to growth, yield and adaptation to biotic or abiotic stress (Li et al., 2014). Conventional digital RGB cameras have been widely used in plant phenotyping platforms since allowing a wide range of phenotyping applications (Araus and Kefauver, 2018). One of the main advantages is the wide versatility of the data (information) collected by RGB cameras, which is essentially linked to the high resolution of these images and the general high quality of factory color calibration. RGB devices have excellent spatial and temporal resolution; they can produce a very large number of images in very short periods, they are portable, and there are many software tools to perform image processing (Perez-Sanz et al., 2017). Although, limitations in RGB-derived information may arise in standardizing the light conditions, the potential impact of light conditions is not necessarily an overwhelming problem because it is often less relevant than expected (e.g., for wheat and barley assessed under Mediterranean conditions; Araus and Kefauver, 2018).

Our work proves that the use of RGB images for the detection of the DB is an effective tool for evaluating the resilience of durum wheat plants to chronic water stress or the effect of biostimulants in supporting tomato plants under water shortage, thus supporting the application of RGB not only for canopy measurements but also for the evaluation of more complex traits. Since this is a parameter easily recordable using RGB images it is possible to implement this instrument in simple field phenotyping platforms or even in UAVs fitted with high quality cameras, provided that solutions for high quality color calibration tools are implemented in an environment with often erratic light quality (Andrade-Sanchez et al., 2014).

Our approach performed under a controlled environment and using a HTP platform has proven to be an excellent approach to select durum wheat genotypes for resilience to water stress.

By using this approach, we have succeeded in identifying a number of genotypes within the landraces collection with potentially increased reliance to DS. Exotic germplasm such as landraces and wild relatives possess high levels of genetic diversity for valuable traits, including adaptation to stressful environments (Wang et al., 2017) traditionally grown and used in the centers of origin and domestication located for durum wheat around the Fertile crescent, and centers of diversification in N. Africa and in the highlands of Ethiopia characterized by challenging environment and water availability (Janni et al., 2018). Finally, our phenomic investigation on tomato plants revealed the ability of different biostimulant prototypes to increase DB and WUE.

This is in accordance with the documented ability of biostimulants to modify physiological processes in plants in a way that provides potential benefits to growth, development or stress response is widely recognised (du Jardin, 2015; Lucini et al., 2015; Povero et al., 2016).

The positive effects of biostimulants on tomato growth, yield and quality have been also considered resulting in a high stability of yields under reduced fertilizers application and upon drought treatment (Petrozza et al., 2014; Koleška et al., 2017), moreover several effects have been proposed (du Jardin, 2015). However, in case of low water regimes the main action feature of biostimulants resides in their high content of aminoacid (Koleška et al., 2017). Furthermore, a strong modification in the gene expression profile and correlated with increased drought adaptation have been demonstrated (Petrozza et al., 2014).

This approach allowed for the selection of 2148 as the most effective prototype, that can be proposed as a new solution able to modulate plant physiology so that plants require less water per unit of yield, and induce optimal plant response under reduced water availability. Such biostimulant-based approach can be adopted to reduce unproductive water losses and maintain healthy, vigorously growing crops for both irrigated and rainfed cropping systems. This should be associated with the choice of well-adapted crop types, together with the optimized management of water, nutrient and agronomic practices. Effective biostimulants like the ones investigated in this study would also allow to reduce the use of irrigation especially in hot, dry environments, where irrigation is most wasteful, in that it produces the least yield per unit of water, as a result of high evaporation rates.

This work provides new evidence in the suitability of RGB images as tool for assessing the WUE for genotype selection as well as and the effects of new biostimulant formulations in limiting environment condition in controlled environment, open also new perspective for its application also in field conditions. The results obtained in our work can be easily exploited in different cultivation systems and transferred to several crops outside the Mediterranean area. The phenomic approach and the proxies identified for physiological response to drought tolerance and biostimulant application to improve water use in crops, can be widely adopted and are of global interest for agriculture sustainability.

## Author Contributions

MJ and DP drafted the manuscript. DD and NB carried out the experiments of growth, treatments and phenotyping activities, and contribute to the data analyses. SS and AP carried out the image analyses and performed the data analyses and interpretations. DDP provided technical assistance. FC, GP, and AS edited the manuscript. MJ and DP coordinated the wheat experiments. GP planned the biostimulants experiments. AS realized the biostimulants formulations. All authors have read and approved the final manuscript.

## Conflict of Interest Statement

The authors declare that the research was conducted in the absence of any commercial or financial relationships that could be construed as a potential conflict of interest.
