# Corrigendum: Can High Throughput Phenotyping Help Food Security in the Mediterranean Area?

**DOI:** 10.3389/fpls.2019.00737

**Published:** 2019-05-31

**Authors:** Donatella Danzi, Nunzio Briglia, Angelo Petrozza, Stephan Summerer, Giovanni Povero, Alberto Stivaletta, Francesco Cellini, Domenico Pignone, Domenico De Paola, Michela Janni

**Affiliations:** ^1^Institute of Biosciences and Bioresources, National Research Council, Bari, Italy; ^2^Dipartimento delle Culture Europee e del Mediterraneo: Architettura, Ambiente, Patrimoni Culturali, Università degli Studi della Basilicata, Matera, Italy; ^3^ALSIA Centro Ricerche Metapontum Agrobios, Metaponto, Italy; ^4^Valagro SpA, Atessa, Italy; ^5^Institute for Veterinary and AgriFood Bioethics, Fiumicino, Italy; ^6^Institute of Materials for Electronics and Magnetism, National Research Council, Parma, Italy

**Keywords:** high throughput phenotyping, digital biovolume, water use efficiency, biostimulants, genetic resources, durum wheat, tomato

In the original article, the reference for the “European Biostimulant Industry Council” was incorrectly written as “(Electron-Beam-Induced Current [EBIC, 2018])”. It should be “(EBIC, 2018).”

The authors apologize for this error and state that this does not change the scientific conclusions of the article in any way. The original article has been updated.
