# Supplementary material for: Can High Throughput Phenotyping Help Food Security in the Mediterranean Area?
Source: Front Plant Sci. 2019 Jan 25;10:15. doi: 10.3389/fpls.2019.00015 (PMC6355677; doi:10.3389/fpls.2019.00015)
Supplement: Supplementary file 1 [file Data_Sheet_1.pdf]

Supplementary Material

Can high throughput phenotyping help food security in the Mediterranean area?

Danzi D[1&]., Briglia N[&2]., Petrozza A[3]., Summerer S[3]., Povero G[4]., Stivaletta A[4]., Cellini F[3]., Pignone D[1,6]., De Paola D[1]., Janni M [1,5*].

*** Correspondence:** michela.janni@ibbr.cnr.it

Supplementary Table 1. Water Use Efficiency (WUE) average values of all the control and drought stressed SSD plants analysed by HTP and results of the ANOVA test.

DAS	Water use efficiency		Treatment	Genotype	G X T
	Control plants	Drought plants			
55	2,9531	2,9973	F=2.166; p=0.143	F=2.033; p<0.001	F=0.713; p=0.888
62	6,4288	6,3469	F=0.005; p=0.941	F=14.507 p<0.001	F=1.063; p=0.387
76	23,7440	23,0281	F=0.993; p=0.321	F=17.534; p<0.001	F=1.122; p=0.307
92	43,7228	41,9908	F=1.943; p=0.166	F=19.878; p<0.001	F=1.784; p=0.009
104	53,1160	50,1078	F=11.849; p<0.001	F=19.007; p<0.001	F=0.826; p=0.749
112	50,2192	42,7207	F=121.141; p<0.001	F=17.719; p<0.001	F=1.158; p=0.266
117	47,6894	39,2731	F=225.627; p<0.001	F=22.647; p<0.001	F=1.375; p=0.094
124	40,9211	34,1989	F=177.026; p<0.001	F=22.914; p<0.001	F=2.048; p=0.001
132	32,7817	27,4107	F=160.174; p<0.001	F=23.608; p<0.001	F=2.485; p<0.001
139	30,3116	23,9930	F=231.910; p<0.001	F=22.817; p<0.001	F=2.272; p<0.001
147	26,3131	21,1851	F=173.620; p<0.001	F=18.456; p<0.001	F=1.997; p=0.002

Supplementary Table 2. WUE ratios for the different genotypes calculated by dividing the WUE values for drought stressed plants by the WUE values for plants in the control group.

	DAS										
**SSD**	**55**	**62**	**72**	**92**	**104**	**112**	**117**	**124**	**132**	**139**	**147**
**35**	1,23	1,12	0,85	1,04	1,12	0,81	0,78	0,86	0,89	0,84	0,82
**44**	1,03	0,93	0,93	1,31	0,84	0,83	0,85	0,85	0,91	0,88	0,85
**64**	1,06	0,97	0,95	0,98	0,81	0,94	0,85	0,94	0,92	0,77	0,74
**69**	0,97	1,10	1,15	1,09	1,07	0,93	0,87	0,89	0,88	0,87	0,77
**92**	1,08	0,93	1,14	0,93	0,83	0,77	0,73	0,73	0,77	0,79	0,72
**96**	0,86	0,98	1,00	0,79	1,12	0,77	0,81	0,97	0,98	0,94	0,93
**99**	1,11	1,03	0,95	1,15	1,10	0,98	0,67	0,64	0,57	0,47	0,48
**109**	1,03	1,08	1,24	1,16	0,96	0,91	0,93	0,91	0,92	0,82	0,85
**112**	1,16	0,99	0,99	1,07	0,86	0,76	0,71	0,74	0,75	0,72	0,75
**116**	0,92	1,01	0,97	0,98	0,86	0,90	0,81	0,83	0,88	0,92	0,89
**122**	1,04	0,85	0,81	0,90	0,79	0,83	0,75	0,71	0,78	0,72	0,67
**135**	0,94	0,85	0,79	0,70	0,79	0,69	0,73	0,72	0,70	0,64	0,63
**171**	0,93	0,95	0,87	0,93	0,89	0,73	0,77	0,81	0,81	0,89	0,90
**178**	1,03	0,76	0,83	0,78	0,92	0,80	0,81	0,83	0,81	0,73	0,76
**195**	1,13	1,19	1,08	1,43	0,96	1,03	0,93	0,93	0,99	0,90	0,95
**231**	0,97	0,92	0,89	0,97	0,91	0,81	0,90	0,97	1,03	0,90	1,07
**244**	1,04	0,97	0,81	0,96	0,80	0,86	0,84	0,95	0,91	0,89	0,88
**253**	0,92	0,90	0,96	0,97	0,92	0,80	0,79	0,75	0,76	0,71	0,67
**269**	0,98	0,84	0,78	0,86	0,96	0,91	0,85	0,81	0,80	0,78	0,76
**278**	1,24	1,19	1,34	1,06	1,13	0,83	0,77	0,72	0,66	0,68	0,72
**322**	1,48	1,17	1,04	1,00	0,94	0,93	0,90	0,90	0,96	0,82	0,86
**325**	1,07	1,02	0,96	0,95	0,99	0,83	0,83	0,82	0,80	0,76	0,77
**335**	1,09	1,12	0,97	1,10	0,88	0,84	0,88	0,91	0,83	0,73	0,76
**343**	0,89	0,93	0,91	0,75	0,85	0,87	0,90	1,01	0,99	0,86	1,01
**397**	1,31	1,09	1,08	0,86	1,00	0,83	0,80	0,82	0,75	0,67	0,70
**409**	1,01	1,05	1,01	1,04	0,90	1,01	0,96	0,92	0,99	0,99	0,99
**415**	0,92	0,97	1,03	0,98	0,99	0,76	0,73	0,79	0,83	0,86	0,80

Supplementary Table 2. Continue

	DAS										
**SSD**	**55**	**62**	**72**	**92**	**104**	**112**	**117**	**124**	**132**	**139**	**147**
**416**	1,17	1,17	1,07	0,96	1,02	0,85	0,89	0,90	1,01	0,96	1,00
**441**	1,41	1,30	1,23	0,93	0,97	0,92	0,88	0,89	0,72	0,69	0,67
**451**	1,05	1,04	1,19	1,11	0,96	0,84	0,78	0,79	0,81	0,85	0,87
**459**	0,98	0,84	0,92	0,83	0,88	0,79	0,80	0,76	0,71	0,61	0,58
**459**	0,98	0,84	0,92	0,83	0,88	0,79	0,80	0,76	0,71	0,61	0,58
**467**	1,11	0,87	0,99	1,04	1,03	0,88	0,82	0,70	0,74	0,74	0,76
**477**	1,32	1,09	1,08	1,08	1,03	0,86	0,88	0,87	0,89	0,85	0,84
**487**	0,86	1,01	1,01	0,97	0,94	0,83	0,79	0,82	0,80	0,77	0,82
**494**	1,10	1,00	1,07	0,96	0,98	0,89	0,89	0,90	0,91	0,87	0,83
**511**	0,99	0,79	0,96	0,77	1,01	0,89	0,81	0,83	0,80	0,71	0,74
**CAPPELLI**	0,76	1,07	0,87	0,86	1,02	0,94	0,93	1,01	1,06	0,96	0,98
**SARAGOLLA**	0,91	1,09	0,98	1,08	0,97	0,79	0,68	0,68	0,70	0,67	0,71
**SVEVO**	0,84	0,83	0,76	0,66	0,97	0,99	0,99	0,96	0,94	0,84	0,84
